# Induction of stress granules alleviates programmed cell death induced by lysosomal damage during NK cell cryopreservation

**DOI:** 10.1038/s41420-026-03149-0

**Published:** 2026-05-07

**Authors:** Yang Liu, Xiaolong Liu, Guangyuan Wu, Xin Wang, Kuo Yu, Hailong Wang, Xin Sun, Zhongyao An, Hongrui Xu, Linlin Zhao, Ce Shi, Song Guo Zheng, Mingli Huang, Zhiren Zhang, Zhenkun Wang

**Affiliations:** 1 NHC Key Laboratory of Cell Transplantation, Harbin Medical University, Harbin, China; 2 Central Laboratory, First Affiliated Hospital, Harbin Medical University, Harbin, China; 3 Key Laboratory of Animal Cellular and Genetics Engineering of Heilongjiang Province, College of Life Science, Northeast Agricultural University, Harbin, China; 4 Obstetrical Department, First Affiliated Hospital, Harbin Medical University, Harbin, China; 5 Department of Child Dental, First Affiliated Hospital, Harbin Medical University, Harbin, China; 6 Department of Blood Transfusion, First Affiliated Hospital, Harbin Medical University, Harbin, China; 7 Department of Immunology, School of Cell and Gene Therapy, Songjiang Research Institute, Shanghai Songjiang District Central Hospital, Shanghai Jiaotong University School of Medicine, Shanghai, China; 8 Department of Cardiology and Pharmacy and Breast Cancer Surgery, Harbin Medical University Cancer Hospital, Institute of Metabolic Disease, Heilongjiang Academy of Medical Science, Heilongjiang Key Laboratory for Metabolic Disorder and Cancer Related Cardiovascular Diseases, Harbin, China

**Keywords:** Immune cell death, Cell death, Immunotherapy

## Abstract

Natural killer (NK) cell-based therapies are under assessment for the treatment of various cancers due to their intrinsic ability to distinguish between malignant and healthy cells in an allogeneic context, enabling off-the-shelf manufacturing possibilities. However, cryopreservation reduces both the recovery and function of NK cells, thereby limiting their therapeutic feasibility. In this study, we evaluated three cryoprotectants (CryoStor 10; ZKCELL FM-01; FBS + DMSO) for the cryopreservation of NK cells. Post-thaw viability, ATP levels, and cytotoxicity were assessed and found to have persistent differences between cryopreserved and fresh cells. Transmission electron microscopy, flow cytometry, and Western blot analysis revealed a complex mode of cell death in cryopreserved cells, which could be partially mitigated by adding some death inhibitors. We further investigated the effects of centrifugation on thawed cells, identifying lysosomal stability as a key determinant of cell death. Pretreatment with low-dose LLOMe prior to cryopreservation induced stress granule formation, stabilizing lysosomes and improving cell recovery rates without compromising effector functional capacity. These findings offer new insights for optimizing NK cell cryopreservation and facilitating their clinical application.

## Introduction

Natural killer (NK) cells are innate immune effector cells involved in the first line of defense against viral infections and malignancies [1–4]. Given their intrinsic antitumor activity and capacity to selectively spare healthy cells in an allogeneic context, the adoptive transfer of donor- and pluripotent stem cell-derived NK cells is an attractive strategy for cancer therapy [5, 6]. Moreover, advances in genetic engineering have facilitated the development of NK cell therapeutics with enhanced antitumor efficiency and resistance to the immunosuppressive tumor microenvironment in both hematological and solid tumors [7–10].

Nevertheless, geographical constraints and stringent storage requirements are two major factors hindering the widespread clinical application of NK cell-based therapies. Cryopreservation provides a feasible approach for long-term storage and time-flexible logistical capacities, but the only comparative clinical report to date showed that cryopreserved NK cells undergo profound cell death within 24 h after thawing, exhibit little activity without interleukin-2 (IL-2) stimulation, and rarely expand in patients [11, 12]. Therefore, it is crucial to optimize cryopreservation protocols that can maintain high viability and functionality of NK cells after thawing, and to thoroughly investigate the mechanisms underlying cryopreservation-induced cellular damage.

It has been proposed that lysosomes may play a major role in the events leading to cellular death [13, 14]. Notably, NK cells have distinct cytoplasmic granules known as secretory lysosomes, a category of secretory vesicles that contain multiple specialized cytolytic proteins along with hydrolases such as cathepsins and granzymes [15]. During the cryopreservation process, organelle membranes can be damaged by intracellular ice crystals [16, 17]. Once the lysosomal membranes were damaged, a wide variety of lytic proteins and hydrolytic enzymes are released, triggering a series of cell death processes [18, 19]. It offers a convincing explanation for why cryopreserving NK cells is more challenging than preserving other cell types.

In recent years, stress granules (SGs) have been shown to condense rapidly at sites of endolysosomal damage, where they enable endomembrane stabilization and repair, prevent further fragmentation and leakage of cellular contents [20, 21]. However, it remains unclear whether SGs play a role in protecting NK cells from cryoinjury. In this study, we observed highly complex modes of cell death in NK cells following freezing and thawing. We hypothesize that pre-activate SGs formation aids in lysosomal repair following freeze-thaw damage, thereby reducing the impact of cryopreservation on NK cells. Low-dose, short-term L-leucyl-L-leucine methyl ester (LLOMe) treatment was employed to enhance NK cell activity following freezing and thawing. We now provide the first evidence that inducing the formation of SGs can enhance lysosomal stability, thereby reducing NK cell death caused by secretory lysosome damage following cryopreservation.

## Results

### Recovery rate and function after cryopreservation with different cryomedia

**Fig. 1:**
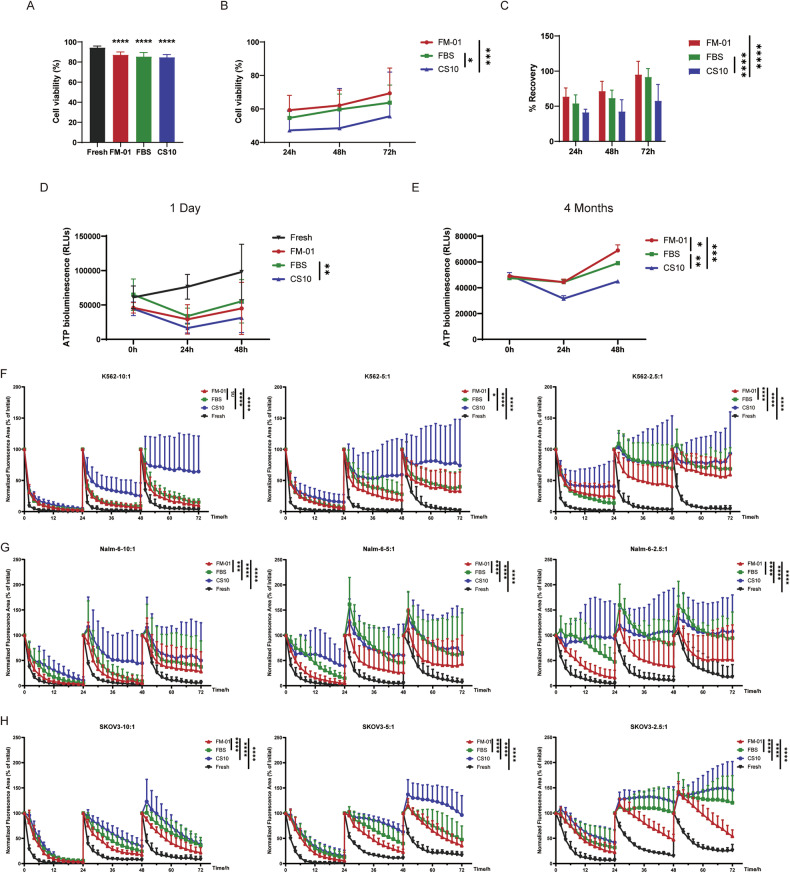
Effects of cryopreservation using three different cryomedia on NK cell viability and function. NK cell viability at 0 h (**A**) and at 24, 48, and 72 h (**B**) after thawing assessed by trypan blue exclusion. The NK cells were cryopreserved for 1 day before thawing (*n* = 12). **C** NK cell recovery at 24, 48, and 72 h after thawing in comparison to starting NK cell number at 0 h (*n* = 9). NK cell ATP levels after 1 day (**D**) or 4 months (**E**) of cryopreservation were assessed using Cell Titer-Glo® over a 48 h period (*n* = 10). NK cytotoxicity assay before and after cryopreservation with K562 (**F**) Nalm-6 (**G**) and SKOV3 (**H**) cells for 72 h under varying effector-to-target (E:T) ratios (*n* = 9). Data are provided as mean ± standard deviation (SD) and were analyzed using one-way analysis of variance (ANOVA) (**A**) and two-way ANOVA (**B**–**H**) with Tukey’s multiple comparisons test.

We first characterized the effects that cryopreservation has on the viability, recovery and function of NK cells using three different cryomedia: CryoStor 10, ZKCELL universal cell freezing medium (FM-01), and FBS supplemented with 10% dimethyl sulfoxide (DMSO). Compared with fresh cells, the viability of all cryopreserved groups declined significantly at 0 h post-thaw, and no significant differences in cell viability were observed among the three cryomedia (FM-01: 87.17% ±8.80%, FBS: 85.45% ±12.90%, CS10: 84.73% ±9.15%) (Fig. 1A). A drastic loss of NK cell numbers accompanied by a significantly reduced cell viability was observed at 24 h post-thaw (Fig. 1B, C). However, between 24 and 72 h post-thaw, cell viability gradually recovered, and significant differences emerged, with FM-01 and FBS groups showing higher viability than the CS10 group (Fig. 1B). Consistently, CellTiter-Glo luminescence-based assays demonstrated that intracellular ATP levels increased from 24 to 48 h post-thaw, with metabolic activity in the FBS groups significantly exceeding that of the CS10 group after one day of storage (Fig. 1D). After four months of storage, FM-01-preserved cells maintained higher ATP levels at 24 and 48 h post-thaw (Fig. 1E).

Although GzmB and IFN-γ levels were lower in cryopreserved NK cells than in fresh cells, these differences were statistically non-significant, except for a significant decline in IFN-γ expression within the CS10 group (Supplementary Fig. 1). Flow cytometry analysis revealed similar expression of surface markers NKG2D, NKG2A, NKG2C, NKp30 and CD94 between cryopreserved and fresh cells. However, we observed a significant loss of NKp46 and CD69 expression in cryopreserved cells after thawing (Supplementary Fig. 2), indicating impaired NK cell activation which may compromise their cytotoxic activity.

To further evaluate effector function, cells from each group were co-cultured with tumor cells at E:T ratios of 10:1, 5:1, and 2.5:1. Against both hematological and solid tumor cell lines, a consistent trend was observed: the cytotoxic activity of the three groups of cryopreserved cells was significantly lower than that of the fresh group, with FM-01 showing better cytotoxic effects than the other two groups (Fig. 1F–H). In summary, although different cryoprotective solutions showed varying effects on NK cell cryoinjury, all three groups experienced a sharp decline in viability within 24 h, indicating that cryoinjury to NK cells remains unavoidable.

### Analysis of the modes of death and subcellular damage of NK cells after thawing

**Fig. 2:**
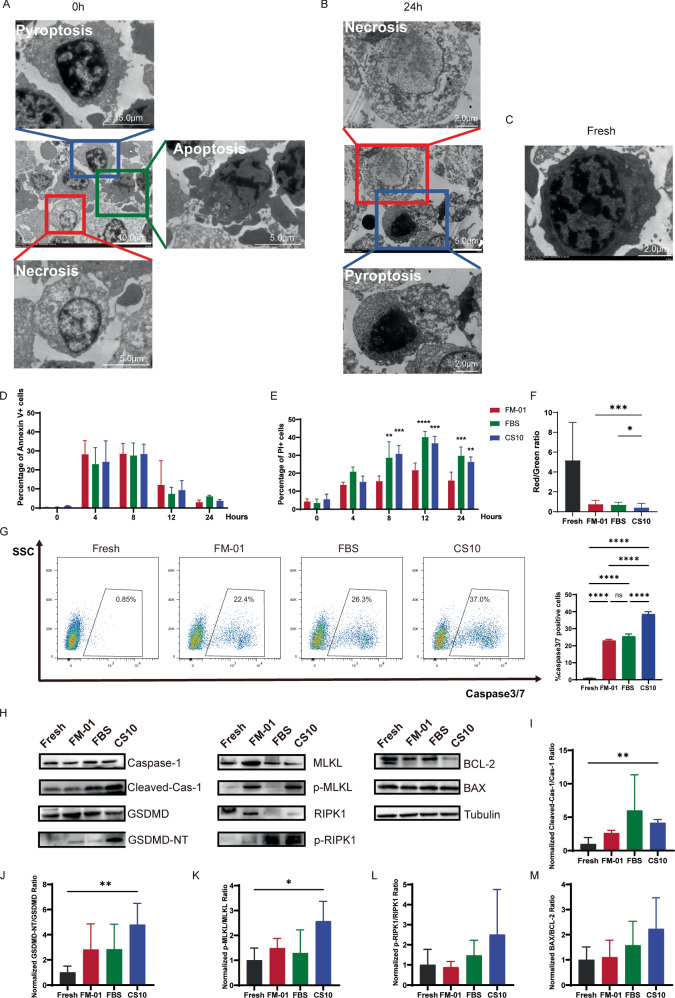
Cell cryopreserved triggers multiple modes of cell death. Representative TEM images of NK cell morphology after thawing 0 h (**A**) and 24 h (**B**). **C** The TEM images of fresh NK cells. The NK cell apoptosis (**D**) and necrosis (**E**) after freezing in different cryomedia were measured using flow cytometry (*n* = 3). **F** Mitochondrial membrane potential was assessed immediately after thawing by JC-1 red/green fluorescence ratio. JC-1 monomer: green; JC-1 aggregate: red (*n* = 6). **G** Flow cytometric assay kit used to detect caspase-3/7 activity immediately after thawing in NK cells cryopreserved in three different media (*n* = 3). **H** Western blot and quantitative analysis showed the expression levels of caspase-1, cleaved caspase-1, GSDMD, GSDMD-NT, MLKL, phosphorylated MLKL, RIPK1, phosphorylated RIPK1, BAX and BCL-2, in NK cells frozen by different cryomedia. **I**–**M** The semi-quantitative analysis of the data in panel (**H**) was performed using Image J software. Data are presented as fold changes relative to the fresh group (*n* = 3). Data statistics are presented as mean ± SD and were analyzed using one-way ANOVA with Tukey’s multiple comparisons test (**F**, **G**), two-way ANOVA (**D**, **E**) with Tukey’s multiple comparisons test and unpaired Student’s *t* test (**I**–**M**).

To investigate the mechanisms underlying cryoinjury, the ultrastructural observations of NK cells after thawing were conducted using transmission electron microscopy (TEM). Multiple types of cell death were observed in NK cells after thawing, with different modes of cell death occurring at various time points. Morphological features of apoptosis, necrosis, and pyroptosis were observed at 0 h, while necrosis and pyroptosis predominated by 24 h (Fig. 2A–C). Then the cells were stained with propidium iodide (PI) and annexin V in order to quantify the apoptotic and necrotic cell populations. It shows that NK cells initially exhibit low levels of both apoptosis (annexin V^+^) and necrosis (PI^+^). However, between 4 and 8 h, there is a significant increase in both apoptosis and necrosis levels, with necrotic cells becoming predominant by 12–24 h (Fig. 2D, E and Supplementary Fig. 3A). Our results also demonstrate that NK cells undergo marked damage within 4–12 h after thawing. Previous studies assessing NK cell function and phenotype following cryopreservation have primarily relied on immediate post-thaw evaluations, which likely overestimate their viability and functional capacity.

The results drove us to gain insights into the key mediators of lytic cell death. We first assessed the mitochondrial membrane potential (MMP) by measuring JC-1 fluorescence. The red fluorescence indicates the presence of JC-1 polymers within the mitochondrial membrane, while the green fluorescence represents JC-1 monomers outside the membrane. The JC-1 ratio, defined as the ratio of red to green fluorescence intensity, reflects the level of MMP. Our results revealed a significant decrease in the JC-1 ratio in NK cells after thawing compared to the fresh group, indicating a substantial reduction in MMP (Fig. 2F). This decline in MMP is recognized as an early manifestation of mitochondrial damage. Freeze-thawed NK cells also showed a marked increase in activation signal from CellEvent™ Caspase-3/7 (Fig. 2G). In addition, western blot analysis on phosphorylated MLKL and phosphorylated RIPK1 (molecules involved in necroptosis [22–24]), cleaved caspase-1 (a molecule primarily involved in pyroptosis), GSDMD (a key executioner of pyroptosis [25, 26]), BAX and BCL-2 (molecules involved in apoptosis [27]) indicated that the activation of programmed cell death pathways involves apoptosis, necroptosis and pyroptosis (Fig. 2H–M). Notably, in addition to ice crystal-induced non-programmed necrosis, phosphorylation of necroptosis-related proteins RIPK1 and MLKL was markedly increased, confirming that freezing stress can simultaneously trigger both non-programmed and programmed necrosis. Overall, the cell death resulting from cryoinjury involves highly complex molecular mechanisms.

### Cryoprotective effect of co-treatment with death inhibitors on NK cells

**Fig. 3:**
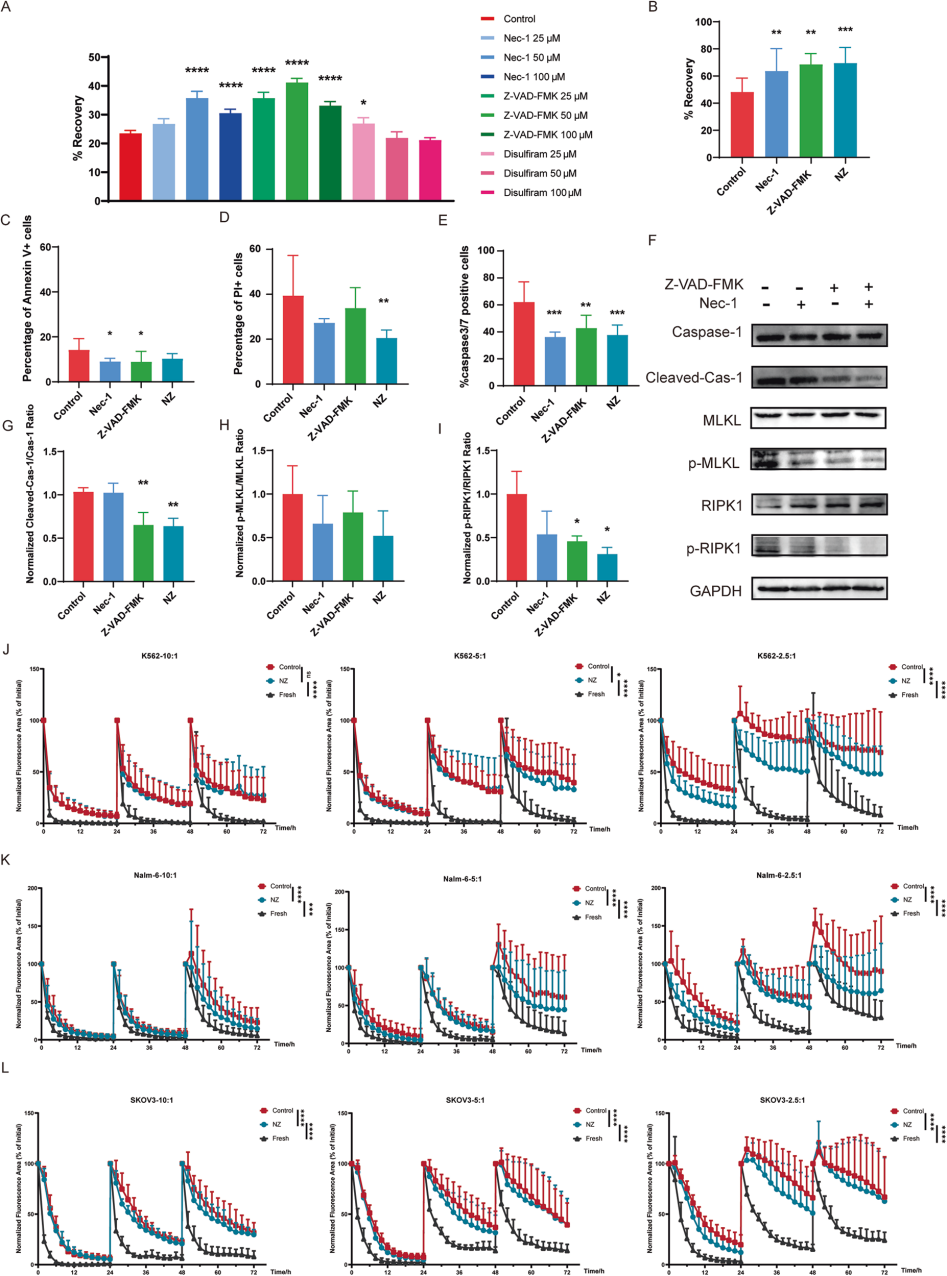
Improved cryopreservation with death inhibitor supplementation. **A** Recovery of NK cells cryopreserved with different concentrations of Necrostatin-1 (NEC-1), Z-VAD-FMK, Disulfiram (*n* = 4). **B** Recovery of NK cells cryopreserved in cryomedia containing NEC-1, Z-VAD-FMK and their combination, measured at 24 h post-thaw (*n* = 12). Apoptosis (**C**) and necrosis (**D**) after being frozen with cryomedia containing different inhibitors measured by flow cytometry (*n* = 8). **E** Caspase-3/7 activity in NK cells was assessed using a Caspase-3/7 Assay Kit for flow cytometry after being frozen with cryomedia containing different inhibitors (*n* = 4). **F** Western blot analysis showed the expression levels of caspase-1, cleaved caspase-1, MLKL, phosphorylated MLKL, RIPK1, and phosphorylated RIPK1 in NK cells (*n* = 3). **G**–**I** The semi-quantitative analysis of the data in panel (**F**) was performed using Image J software. Data are presented as fold changes relative to the Control group. Live-cell imaging (Cellcyte X) was used to evaluate the cytotoxicity of fresh NK cells, NK cells cryopreserved in FM-01 and inhibitors-supplemented cryomedia co-cultured under different E:T ratios with K562 (**J**) Nalm-6 (**K**) and SKOV3 (**L**) cells for 72 h (*n* = 9). Data statistics are presented as mean ± SD and were analyzed using one-way ANOVA (**A**–**E**, **G**–**I**) and two-way ANOVA (**J**–**L**) with Tukey’s multiple comparisons test.

Based on the analysis of cell death pathways shown in Fig. 2, we supplemented the cryomedia FM-01 with the pan-caspase inhibitor Z-VAD-FMK, which blocks apoptosis and pyroptosis [28, 29]; necroptosis inhibitor NEC-1, targeting RIPK1 kinase activity [30]; and pyroptosis inhibitor Disulfiram which blocks GSDMD-mediated pore formation [31]. To examine the efficiency of NEC-1, Z-VAD-FMK, or Disulfiram at different concentrations on NK cell cryopreservation, we evaluated recovery rate after cryopreservation and culture. Our results indicated that Disulfiram slightly improve recovery relative to the FM-01 control group, whereas supplementation with 50 μM Z-VAD-FMK (41.13 ± 1.493%) or NEC-1 (35.75 ± 2.363%) significantly enhanced recovery compared with the control (23.50 ± 1.000%) (Fig. 3A). Therefore, we chose 50 μM Z-VAD-FMK or/and 50 μM NEC-1 as the optimal concentration for the effective cryopreservation of NK cells and used this in all subsequent experiments.

Similarly, we observed that Z-VAD-FMK, NEC-1, and their combination (NZ group) all enhance the recovery of NK cells (Fig. 3B). Notably, necrosis was significantly reduced in the combination group after thawing 8 h (Fig. 3C, D). To further evaluate the beneficial effects of the two inhibitors in reducing cryoinjury, we conducted a caspase-3/7 activity assay and performed Western blot analysis on p-RIPK1, cleaved caspase-1, and p-MLKL. Flow cytometry analysis revealed that caspase-3/7 activity was significantly reduced in the three inhibitor-treated groups compared to the FM-01 control group (Fig. 3E). Compared to the FM-01 control, the three inhibitor-treated groups all exhibited significantly reduced p-RIPK1 and cleaved caspase-1 expression in cryopreserved NK cells. We also observed a modest decrease in p-MLKL expression although these changes did not reach statistical significance (Fig. 3F–I).

Although NEC-1 or Z-VAD-FMK alone partially attenuated cell death, combined treatment with both NEC-1 and Z-VAD-FMK resulted in a more pronounced effect, as evidenced by a more complete suppression of p-RIPK1 and caspase activation. Furthermore, in serial killing assays using the Cellcyte X system, NK cells in the NZ group exhibited significantly enhanced killing of K562, Nalm-6, and SKOV3 tumor cells compared with the FM-01 control group (Fig. 3J–L).

### Centrifugation of cells after thawing triggers cell death and lysosomal rupture

**Fig. 4:**
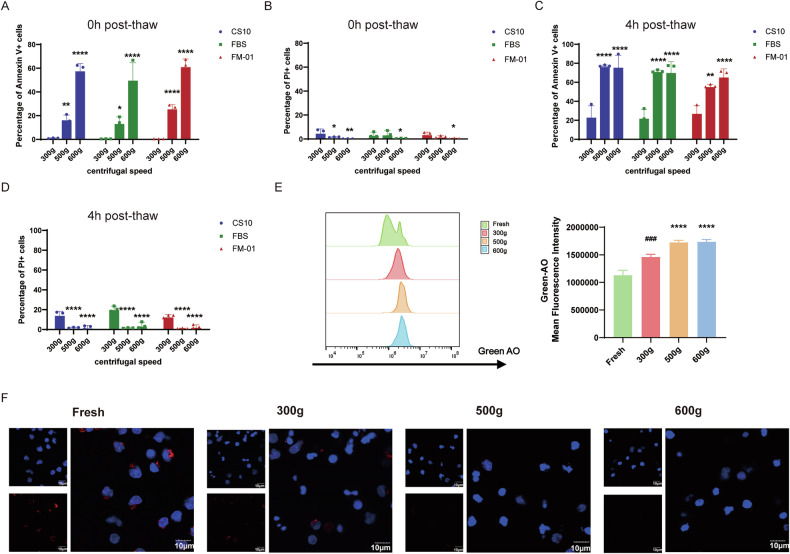
Centrifugation of cells after thawing triggers cell death and lysosomal rupture. Apoptosis (**A**) and necrosis (**B**) levels in thawed NK cells cryopreserved in three cryomedia at varying centrifugal speeds (*n* = 3). Apoptosis (**C**) and necrosis (**D**) levels 4 h post-thaw in NK cells cryopreserved in three cryomedia at varying centrifugation speeds (*n* = 3). **E** Quantitative analyses of the green fluorescence intensity of acridine orange (*n* = 4), ^****^P < 0.0001 versus the fresh group, ^###^*P* < 0.001 versus the 500×*g* group. **F** Following thawing, NK cells were immediately centrifuged at various speeds and then incubated with LysoTracker Red. Data statistics are presented as mean ± SD and were analyzed using two-way ANOVA (**A**–**D**) and one-way ANOVA (**E**) with Tukey’s multiple comparisons test.

Due to the toxicity of DMSO, washing by centrifugation is required both to remove DMSO and serum in experimental studies and to minimize infusion-related toxicity in clinical applications [6, 32]. Firstly, we compared diluted and undiluted samples before centrifugation, and found no statistically significant differences, indicating that prompt processing makes DMSO toxicity not the main factor causing NK cell damage (Supplementary Fig. 4). To further investigate the effect of Relative Centrifugal Force (RCF) on thawed cell viability, we compared cell death and recovery rates at different centrifugation speeds. The results demonstrated that as RCF increase, the cell death rate detected immediately after thawing rises, with apoptosis showing the most pronounced increases (Fig. 4A, B). However, centrifugation at 300–600 × *g* does not have any significant adverse effects on fresh NK cells (Supplementary Fig. 3B, C). Notably, the dependence of cell death on centrifugation parameters was more evident at 4 h post-thaw compared with immediately after thawing (Fig. 4C, D), suggesting that centrifugation-induced death is mediated not only by mechanical stress but also by programmed cell death pathways. Furthermore, we performed AO staining to detect lysosomal destabilization [33]. Higher centrifugation enhanced green fluorescence in NK cells as dye escaped and bound with nucleic acids (Fig. 4E). We also used LysoTracker Red dye to stain NK cells, which showed decreased fluorescence intensity under higher centrifugation parameters, further indicating aggravated lysosomal damage (Fig. 4F).

### LLOMe pretreatment improves recovery and function of NK cells after cryopreservation by inducing SGs formation and lysosomal stability

As shown in Supplementary Fig. 5, cryopreserved NK cells exhibited a loss of LysoTracker fluorescence signal compared to the fresh group. And the cryopreservation process enhanced green fluorescence in AO-stained cells. These findings collectively indicate substantial lysosomal damage in cryopreserved cells relative to fresh cells.

**Fig. 5:**
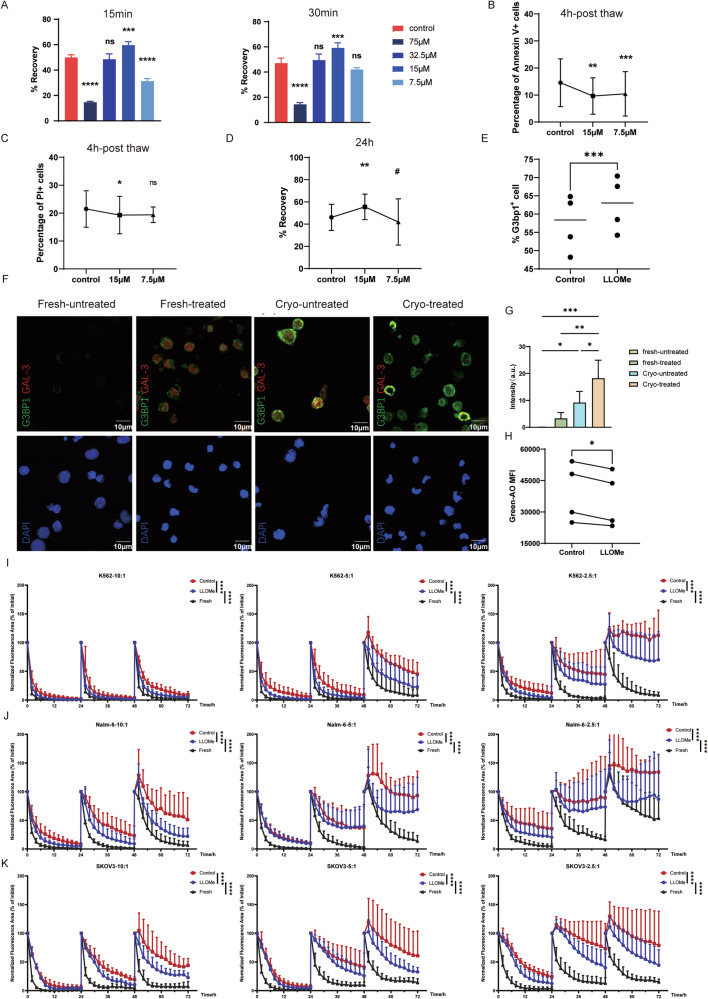
Repair of lysosomal rupture improves cryopreservation. **A** NK cell recovery treated with LLOMe (15 or 30 min) at final concentrations of 75 μM, 32.5 μM, 15 μM and 7.5 μM after thawing 24 h (*n* = 4). Apoptosis (**B**) and necrosis (**C**) were assessed 4 h post-thaw in thawed NK cells pretreated with two concentrations of LLOMe (*n* = 8). **D** Recovery of NK cells treated with various concentrations of LLOMe at 24 h post-thaw (*n* = 9), ^**^P < 0.01 versus the control group, ^#^*P* < 0.05 versus the 15 μM group. **E** Flow cytometry analysis of G3BP1 expression in thawed NK cells at 0 h post-thaw (*n* = 4). **F** NK cells were left untreated or treated with LLOMe (15 μM, 30 min) and stained for G3BP1 and GAL-3. **G** Quantitative analysis of G3BP1 fluorescence intensity (*n* = 4). **H** Quantitative analysis of the green fluorescence intensity of acridine orange (*n* = 4). **I**–**K** Live-cell imaging (Cellcyte X) was used to evaluate the cytotoxicity of fresh NK cells, LLOMe-pretreated and untreated NK cells after cryopreservation co-cultured under different E:T ratios with K562 (**I**) Nalm-6 (**J**) and SKOV3(**K**) cells for 72 h (*n* = 9). Data statistics are presented as mean ± SD and were analyzed using one-way ANOVA (**A**–**D**, **G**) with Tukey’s multiple comparisons test, unpaired Student’s *t* test (**E**, **H**) and two-way ANOVA (**I**–**K**) with Tukey’s multiple comparisons test.

**Fig. 6:**
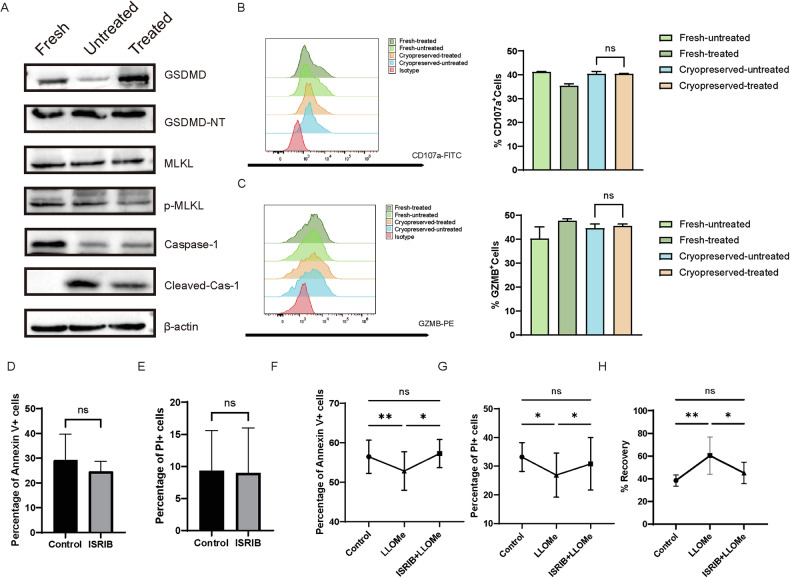
LLOMe improves NK cell cryopreservation via stress granule-dependent lysosomal repair. **A** Western blot analysis showed the expression levels of caspase-1, cleaved caspase-1, MLKL, phosphorylated MLKL, GSDMD and GSDMD-NT in NK cells from the following groups: fresh NK cells, untreated NK cells cryopreserved with FM-01 and LLOMe-treated NK cells cryopreserved with FM-01. Flow cytometry assay for CD107a (**B**) and GzmB (**C**) levels in fresh and cryopreserved NK cells with or without pretreatment with 15 μM LLOMe for 30 min (*n* = 2). Flow cytometric detection of apoptosis (**D**) and necrosis (**E**) levels in ISRIB-treated and untreated NK cells (*n* = 3). Apoptosis (**F**) and necrosis (**G**) levels in NK cells with different pretreatments after thawing 4 h (*n* = 5). **H** Recovery of cryopreserved NK cells with different pretreatment after thawing 24 h (*n* = 8). Data statistics are presented as mean ± SD and were analyzed using unpaired Student’s *t* test (**D**, **E**) and one-way ANOVA (**B**, **C**, **F**–**H**) with Tukey’s multiple comparisons test.

To address this, we sought to promote lysosomal repair by enhancing SGs formation through the artificial damage of lysosomes using low-dose LLOMe treatment. First, we optimized the concentration of LLOMe to effectively induce SGs formation without compromising cell recovery (Fig. 5A). By evaluating necrosis and apoptosis, we determined that 15 μM for 30 min was the optimal condition (Fig. 5B, C), a result further validated by trypan blue staining assays of cell recovery (Fig. 5D). Consistent with the reduction in cell death after freezing and thawing, indicators associated with lytic cell death pathways were also downregulated (Fig. 6A).

For quantitative validation, flow cytometry results confirmed the expression of G3BP1 protein was significantly elevated (Fig. 5E), reflecting enhanced SGs formation [34, 35]. Subsequently, immunofluorescence experiments revealed co-localization of the SGs core protein G3BP1 with galectin-3 (GAL-3), a conventional marker for lysosomal damage [20]. NK cells pretreated with LLOMe exhibited SGs recruitment prior to cryopreservation, which became more pronounced after freezing and thawing (Fig. 5F, G). Additionally, flow cytometry assessed lysosomal stability post-thaw. AO staining showed diminished green fluorescence, indicative of improved lysosomal membrane stability and mitigated damage (Fig. 5H). Finally, the cytotoxic activity of both LLOMe-pretreated and untreated NK cells was assessed in killing assays using three tumor cell lines K562, Nalm-6 and SKOV3 as targets, LLOMe-pretreated NK cells displayed markedly enhanced cytotoxic efficiency (Fig. 5I–K). To verify that pretreatment did not impair degranulation, we measured granzyme B and CD107a expression in both pretreated and untreated groups. The results showed no significant differences, indicating that degranulation was maintained (Fig. 6B, C). Thus, we infer that the 15 μM LLOMe effectively induced lysosome-targeting SGs formation without causing substantial cellular damage.

ISRIB has been identified to prevent the formation of SGs induced by the integrated stress response. To further investigate the relationship between SGs and LLOMe-induced effects, we inhibited stress granule formation in NK cells using ISRIB, after confirming that 1 μM ISRIB (1 h treatment) is non-toxic to NK cells (Fig. 6D, E). Pretreating NK cells with ISRIB prior to cryopreservation suppressed SG formation and reversed the beneficial impact of LLOMe, leading to a reduced recovery rate and elevated levels of cell death (Fig. 6F–H). This outcome aligned with our hypothesis that increased SG formation targeting lysosomes serves to mitigate cryopreservation-induced lysosomal damage.

In summary, these findings demonstrate that low-dose, short-term LLOMe pretreatment promotes lysosome-targeting SGs formation, effectively improving post-cryopreservation lysosomal integrity and enhancing NK cell viability and cytotoxic function.

### LLOMe pretreatment or death inhibitor supplementation enhances the in vivo activity of cryopreserved NK cells

**Fig. 7:**
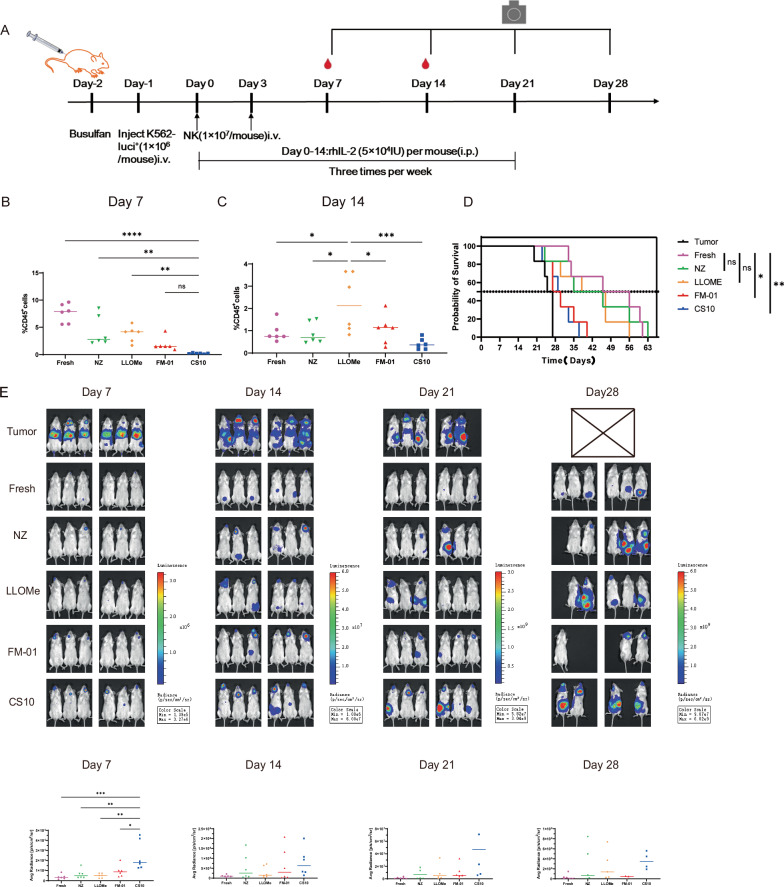
LLOMe pretreatment or death inhibitor supplementation enhances the in vivo activity of cryopreserved NK cells. **A** Schematic diagram of in vivo experiment. **B**, **C** Flow cytometry analysis of the percentage of human leukemic cells (CD45 positive cells) in peripheral blood of treated mice after 7 days (**B**) or 14 days (**C**) of transplantation (*n* = 6 mice/group). **D** Kaplan–Meier survival curves of NK treated mice (*n* = 6 mice/group). The median survival times for the tumor control group, fresh NK group, NZ group, LLOMe group, FM-01 group, and CS10 group were 26 days, 51 days, 40.5 days, 42.5 days, 28.5 days, and 29.5 days, respectively. **E** Luminescent images of treated mice and quantification of tumor burden (flux) (*n* = 6 mice/group). Data statistics are presented as mean ± SD and were analyzed using one-way ANOVA (**B**, **C**, **E**). For survival analysis, survival was estimated using the Kaplan–Meier method, and the log-rank test was used to assess differences between groups.

A key measure of the cryostability of cell therapy products is a comparison of non-cryopreserved versus cryopreserved cells in vivo. To examine this, we generated human leukemia xenograft models by injecting NCG mice with K562 cells transduced with a luciferase expression construct at 1 ×10^6^ cells per mouse. The leukemia-bearing mice were treated with fresh or cryopreserved NK cells in different cryomedia (Fig. 7A). The LLOMe and NZ group cryopreserved NK cells achieved significantly improved tumor control, expansion in the peripheral blood, and survival compared to FM-01 and CS10 cryopreserved NK cells (Fig. 7B–E), indicating that both modified cryopreservation methods can enhance the potency of frozen NK cells.

## Discussion

Despite several decades of NK cell therapy trials, there remain scant reports comparing the activity of fresh versus cryopreserved NK cells in patients [36]. The clinical application of NK cells remains constrained by the limitations of large-scale preparation and long-term preservation technologies [37]. Previous studies have demonstrated that conventional cryopreservation methods often result in reduced viability, impaired function, and abnormal activation of death pathways in NK cells after thawing, severely limiting their clinical accessibility [6, 38–42]. The use of cryopreserved NK cells remains a significant challenge.

To date, the majority of research has concentrated on short-term post-thaw viability and function [11], with limited systematic evaluation of long-term dynamic changes and the impact of cryopreservation solutions on NK cell, which likely leads to overestimation of the true recovery rate. Importantly, our findings indicate that cells undergo significant damage within 4–12 h after thawing, underscoring the importance of monitoring dynamic cellular states over time.

We systematically evaluated the cryopreservation efficacy of three cryoprotectants on NK cells. The results indicated that FM-01 and FBS provided superior short- and middle-term storage (4 months) compared with CS10, as evidenced by higher viability, recovery, ATP levels, and cytotoxic function against both hematologic and solid tumor models, highlighting its potential for establishing clinical NK cell banks. This may be because FM-01 contains human serum albumin, while FBS contains bovine serum albumin, and macromolecules such as serum proteins can maintain cell membrane stability during the procedure, protecting them against cryoinjury [43]. Nevertheless, all cryopreserved groups still exhibited lower viability than fresh NK cells.

Caspase inhibitors, currently used as additive cryoprotectants, have been shown to improve the cryopreservation of sperm [44], oocytes [45], and spermatogonial stem cells [46]. However, multiple studies have reported limited efficacy of the caspase inhibitor Z-VAD-FMK, which is partly due to a portion of cells undergoing secondary death, whereby apoptosis transitions into necroptotic apoptosis [47, 48]. Therefore, we think the combined use of caspase inhibitors and necroptosis inhibitors enhances cell survival following cryopreservation. The potential benefits of this strategy warrant systematic validation.

Additionally, we found that the removal of cryopreservation solution by centrifugation aggravates NK cell cryoinjuries in a speed-dependent manner, mainly manifested as increased apoptosis and exacerbated lysosomal damage. This indicates that post-thaw centrifugation significantly impacts cell viability. We recommend that the centrifugal force does not exceed 300 × *g* for collecting post-thaw NK cells; alternatively, the development of non-centrifugal methods for cryoprotectant removal represents a promising direction to further minimize cell damage.

During slow freezing, ice crystal formation induces membrane disruption and leakage of intracellular components [49, 50], such as lysosomal enzymes, leading to poor post-thaw recovery. In addition, osmotic alterations render thawed NK cells more fragile and susceptible to mechanical stress during centrifugation [51]. Under high RCF, this stress not only aggravates programmed cell death but also compromises lysosomal stability. Besides, pretreatment with IL-15 and IL-18 was demonstrated to significantly improve NK cell recovery after cryopreservation by promoting granzyme B release and upregulating anti-apoptotic genes [12], but this approach reduced NK cell cytotoxicity before cryopreserved.

Given the central role of lysosomal integrity, we explored strategies to repair lysosomes and limit NK cell autolysis. LLOMe induces an increase in lysosomal membrane permeability, leading to the release of Ca²⁺ from the lysosomal calcium pool. Activation of the calcium-dependent ALIX-ALG2 complex subsequently triggers the PKR-PACT-eIF2α phosphorylation pathway. This results in the phosphorylation of eIF2α and triggers global translational arrest in the cell. Subsequently, mRNA-protein complexes (mRNPs) undergo liquid-liquid phase separation (LLPS) with the core RNA-binding protein G3BP1, forming SGs. SGs are targeted to lysosomal damage sites, forming “molecular plugs” that fill membrane pores to prevent leakage of lysosomal contents (e.g., cathepsins) [21, 52]. To address cryopreservation-induced lysosomal leakage caused by cryopreservation, we tested low-dose LLOMe pretreatment to induce SGs. This approach stabilizes lysosomes, prevents granzyme B leakage, reduces cell death, and enhances NK cell-mediated cytotoxicity in tumor co-culture. Importantly, this treatment does not compromise essential effector functions, such as granzyme B release and CD107a expression. Although LLOMe is a potent tool for studying lysosomal damage, its clinical translation is hindered by its cytotoxicity. In the future, cryopreservation could be improved by inducing lysosome-targeting SGs in advance under milder conditions.

**Fig. 8:**
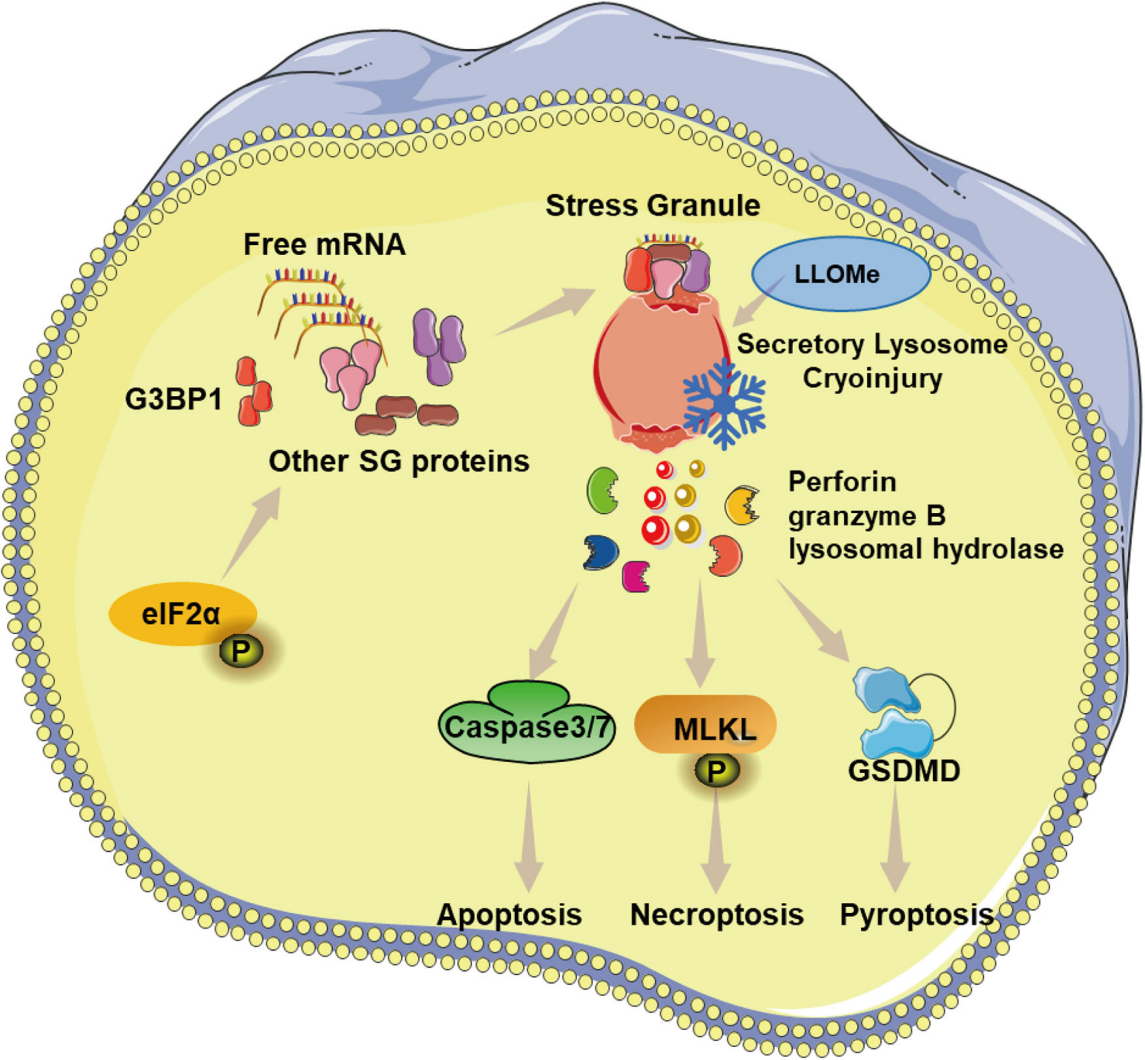
The proposed mechanism by which SG induction mitigates cryoinjury is based on the results of the present study.

In this study, we employed optimized centrifugation parameters, death inhibitors, and LLOMe pretreatment to effectively mitigate the adverse effects of cryopreservation on NK cell viability (Fig. 8). We believe that our findings offer a novel strategy for advancing application of cryopreserved NK cell-based therapies.

## Methods and materials

### Cell line

The K562, Nalm-6 and SKOV3 cell lines used in this study are maintained at the Central Laboratory of the First Affiliated Hospital of Harbin Medical University. All cell lines were recently authenticated by short tandem repeat (STR) profiling, with profiles matching the reference databases.

K562 and Nalm-6 cells were cultured in RPMI-1640 medium (Gibco; 11875093) supplemented with 10% fetal bovine serum (FBS, Sigma; F0193). SKOV3 cells were cultured in Dulbecco’s modified Eagle medium (DMEM; Gibco; 11965118) supplemented with 10% FBS. All the cell lines were cultured in a humidified incubator at 37 °C with 5% CO_2_. All the cell lines used in this study tested negative for mycoplasma contamination.

### NK cell isolation and expansion

NK cells were enriched from blood by Human NK Cell Enrichment Kit (ZKCELL, NC 01-003) according to the manufacturer’s protocol, unwanted cells that conjugate with red cells are removed through density gradient centrifugation using Lymphocyte Separation Medium (ZKCELL, LS01-006). For NK cell expansion, PBMCs were seeding with K562mbIL-21 feeder cells (ZKCELL, NK02-002) at a ratio of 8:1 in Lymphocyte culture medium (ZKCELL, LC01-001) with 5% FBS (Gibco), 200 U/mL IL-2 (SL PHARM). On day 7, add feeder cells equivalent to 1/8 of the total cell numbers again. For expansion, fresh medium was added every 2–3 days for 14 days. Expanded NK cells that achieved at least 90% purity were used for further experiments.

### Inhibitors

Disulfiram, Necrostatin-1 (NEC-1), Z-VAD-FMK, integrated stress response inhibitor (ISRIB) were purchased from MCE.

### Flow cytometry

For membrane staining, 2 ×10^5^ cells were collected and washed with DPBS. Subsequently, the corresponding antibodies were added to the cells, followed by incubation at 4 °C for 30 min. After washing, the cells were then resuspended in 200 μL of DPBS. Flow cytometry (BD, AriaIIu) was utilized to analyze the samples.

For intracellular cytokine staining, 5 ×10^5^ NK cells were plated and treated with BFA (BD ;347688). K562 cells were co-cultured for 5 h to activate NK cells. Following the addition of CD56 antibody, the samples underwent incubation for 30 min. Then, the cells were treated with a fixation solution (BD; 554722) and incubated for an additional 40 min. Post-centrifugation, 200 μL of permeabilization buffer (BD; 554722) was added. Subsequently, the cells were incubated with IFN-γ and Granzyme-B antibodies for 30 min. After two washes with permeabilization buffer, the cells were resuspended in 200 μL of DPBS. Flow cytometry was then employed to analysis the samples.

Detailed information on these antibodies is given in Supplementary Table 1.

### NK cell cryopreservation and thawing

NK cells were harvested from culture, counted, centrifuged, and then resuspended in DPBS. NK cells were frozen in CryoStor CS10 (STEMCELL, 07930), FM-01 (ZKCELL, FM-01) or FBS (10% DMSO + 40% RPMI-1640 + 50% FBS) at a concentration of 1 ×10^8^ cells/mL. The cells were frozen to −80 °C in a CoolCell® cell freezer overnight, followed by transfer to liquid nitrogen for long-term storage. NK cells were thawed in a 37 °C water bath. When only a small piece of ice remained, the cells were either diluted with a 20-fold volume of PBS or centrifuged directly (without dilution) at 300 × *g* for 8 min. Cryomedia was removed and the cells were resuspended in NK cell media at a concentration of 1–2.5 × 10^6^ cells/mL.

### Cell viability by the trypan blue dye exclusion assay

Cell viability of NK cells was determined by the trypan blue assay that depends on quantify live cells by labeling dead cells exclusively. The NK cells were stained with trypan blue dye (0.4% solution) and to count unstained live and blue-stained dead cells under a phase contrast microscope with a hemocytometer.

### Cell Titer-Glo assay

Thawed NK cells were plated in a white, opaque 96-well plates with a final volume of 100 μL of media. The cells were cultured for 0 h, 24 h and 48 h at 37 °C. After incubation, the assay plates were removed from the incubator and allowed to equilibrate to room temperature on the benchtop before the addition of 100 μL of CellTiter-Glo reagent (Promega), following the manufacturer’s instructions.

### Immunofluorescence staining

Cells were seeded on the poly-L-lysine-coated plates fixed by 4% paraformaldehyde, permeabilized, and incubated with antibody. The primary antibody (G3BP1 and Galectin-3) was purchased from Proteintech (1:500, 13057-2-AP, 60207-1-Ig), and secondary fluorescent immunoglobulin G was purchased from Abcam (1:1000, ab150116) and Thermo Fisher Scientific (1:500, A-11008). DAPI staining was used to visualize the nucleus. Pictures were acquired with an Olympus FV3000 microscope.

### Live-cell staining

To assess the lysosome, cells were incubated with LysoTracker Red (100 nM) for 30 min, subsequently incubated with Hochest 33342 (20 μg/mL) for 10 min. For acridine orange (AO) staining, NK cells were incubated with 2 μg/mL of AO for 30 min. Then, the signal was analyzed using a fluorescence microplate reader according to previous methods. Images were captured by an Olympus FV3000 microscope.

### Western blotting

For Western blot analysis, Total protein was lysed in RIPA lysis buffer (Beyotime, P0013B) containing protease inhibitors on ice for 30 min. The lysates were then centrifuged at 12,000 × *g* for 25 min, and the resulting supernatants were collected. Protein concentrations were quantified using the BCA protein assay kit (Beyotime, P0009B). Equal quantities of protein were loaded, separated on a sodium dodecyl sulfate polyacrylamide gel electrophoresis system, and subsequently transferred to a PVDF membrane (Millipore, IPVH00010). After blocking, the membrane was incubated with the appropriate primary antibody (1:1000) overnight at 4 °C. The immunoblots were visualized using HRP-conjugated secondary antibodies (1:7000). The primary antibodies used included: MLKL (Immunoway, YM8455), Phospho-MLKL (CST, 18640), RIPK1 (Immunoway, YM8084), Phospho-RIPK1 (CST, 44590), GSDMD (Abcam, ab210070), GSDMD-NT (Abcam, ab215203), GSDMD-NT (Immunoway, YM8489), BCL-2 (Affinity, AF6139), Caspase-1(CST, 3866), BAX (CST, 5023), GAPDH (Abways, P04406), β-Actin (CST, 3700).

### Cytotoxicity and serial killing assays

NK and target cells that stably expressing a fluorescent protein were co-cultured under comparable conditions at varying E:T ratios in a 96-well plate for three rounds (24 h per round). At the end of each round, target cells were added to the culture plates. The samples were analyzed using the Cellcyte X microscope (Cytena, Germany) along with the associated Cellcyte software.

### In vivo animal models and treatments

NCG mice were purchased from GemPharmatech (Nanjing, China) and maintained in a sterile environment for 1 week to allow for acclimatisation. 24 h after administering Busulfan at a dosage of 10 mg/kg, transplant 1 × 10^6^ K562 cells into the NCG mice (6-8 weeks old, female) via tail vein injection. One day later, six groups of effector cells were injected, with 1 × 10^7^ effector cells administered into the tail vein of each mouse. The effector cells were administered in the same manner and dosage on the third day. Mice were injected with D-luciferin (Gold Biotechnology, USA) at a dose of 150 mg/kg on day 7, day 14, day 21, and day 28, and imaged using PerkinElmer IVIS Lumina LT. Protocols were approved by the Harbin Medical University Animal Care and Use Committee guideline. Data collection and analysis were performed by researchers blinded to the experimental groups, and samples were processed in a randomized order.

### Statistical analysis

Statistical analyses were conducted using GraphPad Prism version 9.5.1. The significance of differences between two groups was assessed with an unpaired Student’s *t* test, while comparisons involving more than two groups were performed using one-way ANOVA. Two-way ANOVA analysis was employed for the statistical analysis of multifactor variance. Significance levels are indicated as follows: ^*^and ^#^*p* < 0.05, ^**^and ^##^*p* < 0.01, ^***^and ^###^*p* < 0.001, ^****^and ^####^*p* < 0.0001 and ns for *p* > 0.05.

## Supplementary information

Uncropped western blots

Supplementary Materials-cddisc

## Data Availability

The datasets generated or analyzed during the current study are available from the corresponding author on reasonable request.
