# Supplementary figures and images for: Induction of stress granules alleviates programmed cell death induced by lysosomal damage during NK cell cryopreservation
Source: Cell Death Discov. 2026 May 7;12:286. doi: 10.1038/s41420-026-03149-0 (PMC13319456; doi:10.1038/s41420-026-03149-0)

Fig. 2


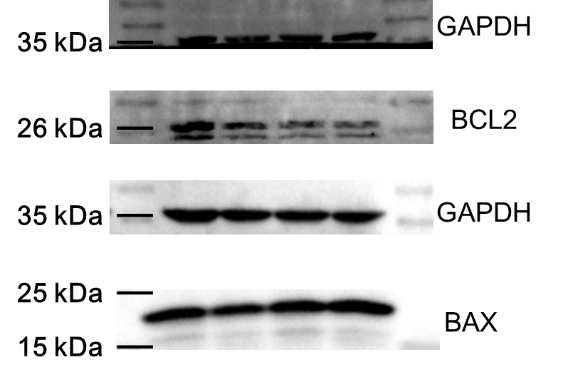

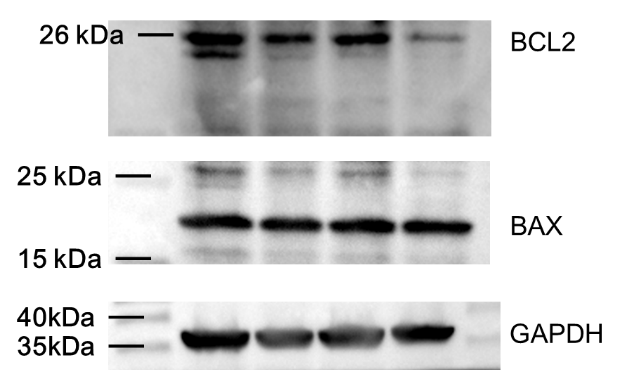


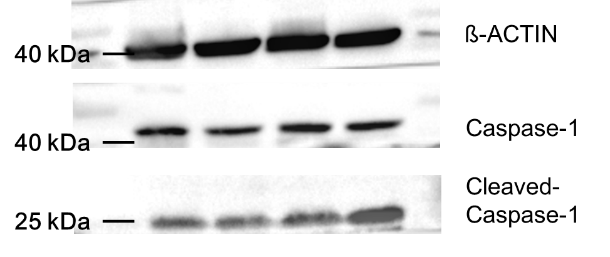

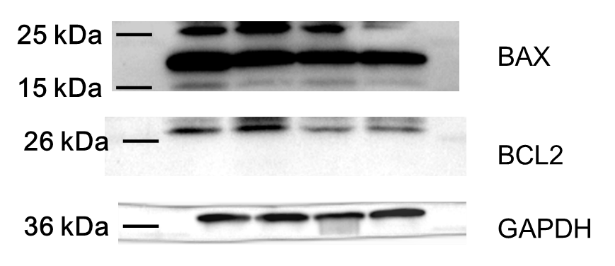


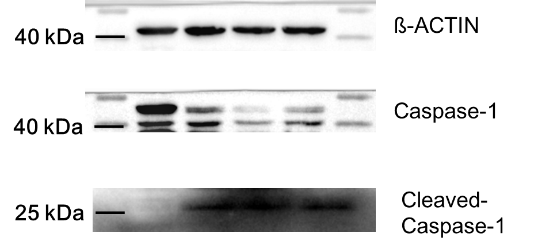

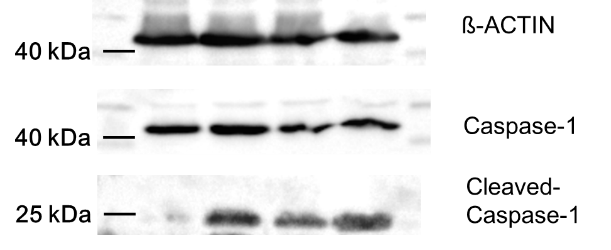


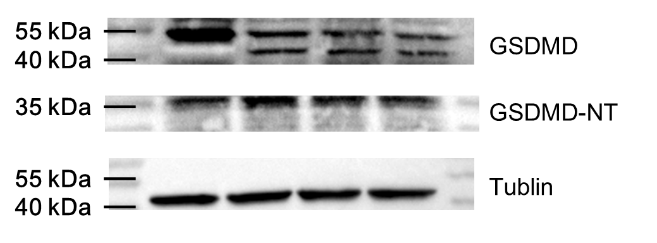

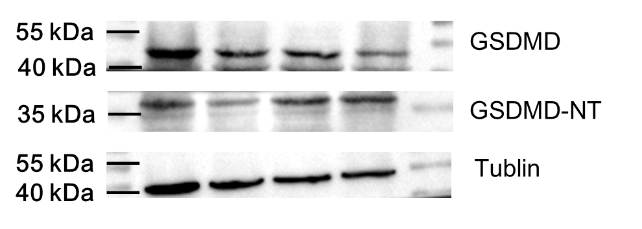


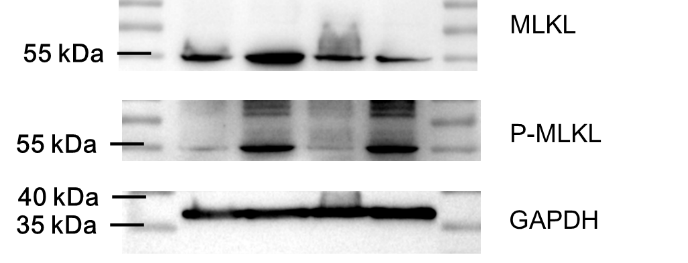

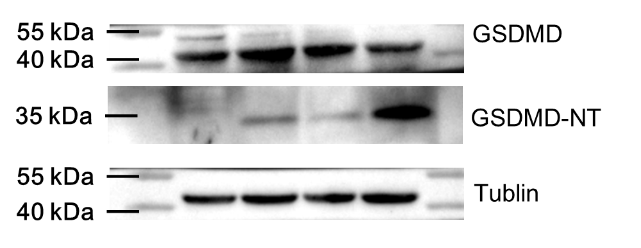


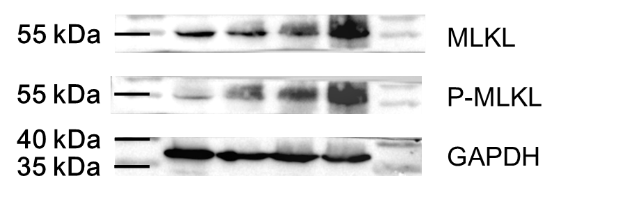


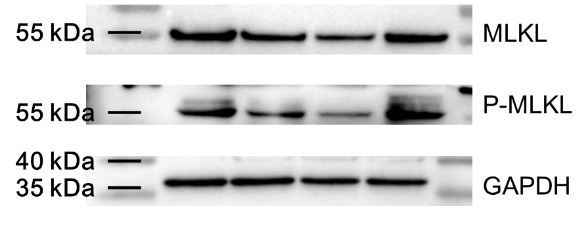


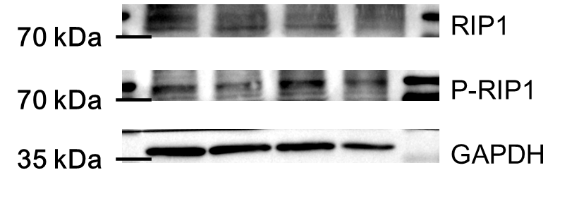


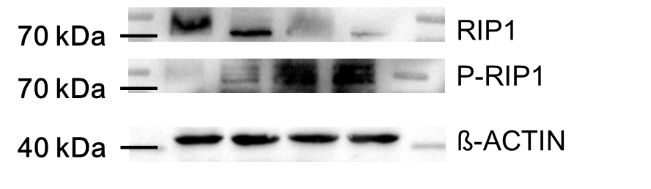

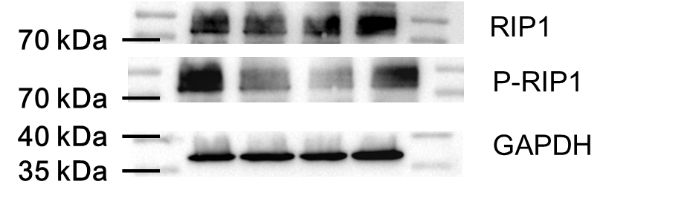


Fig. 3


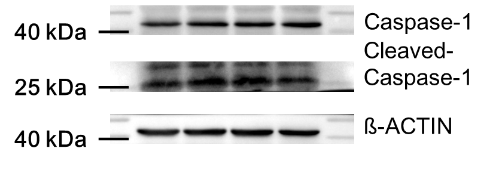

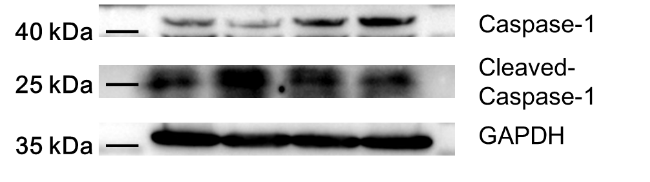


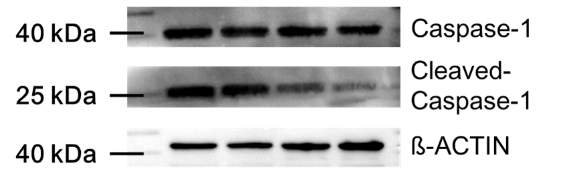

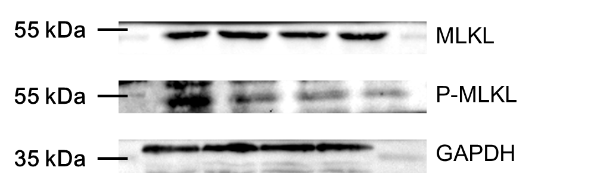


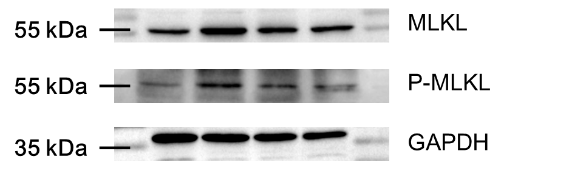

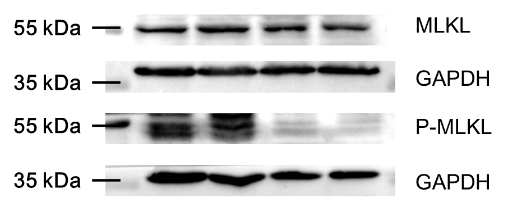


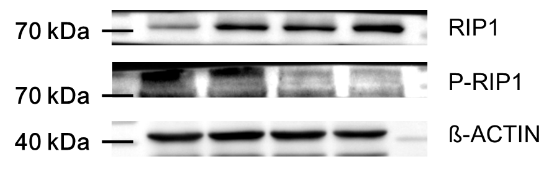

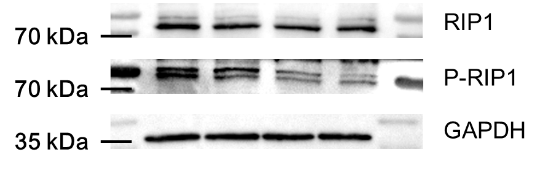


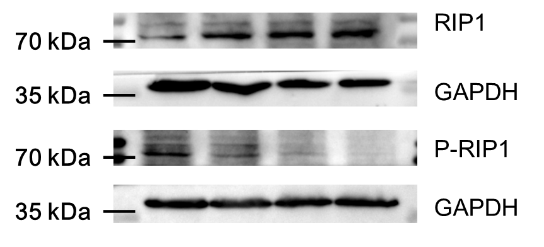


Supplementary Fig. 5D


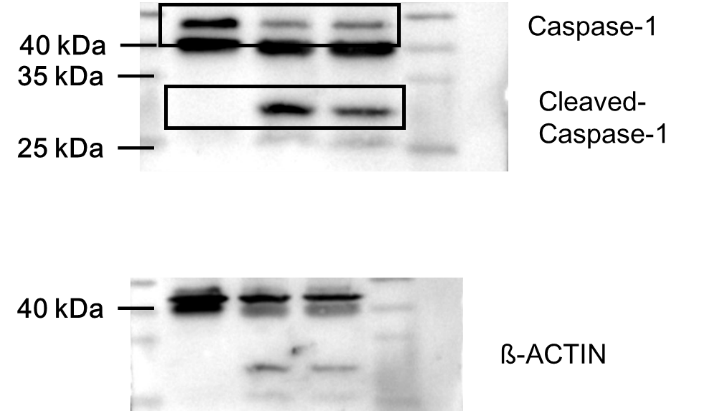


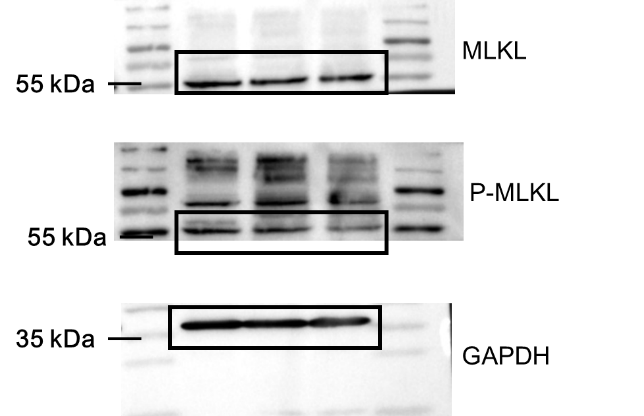


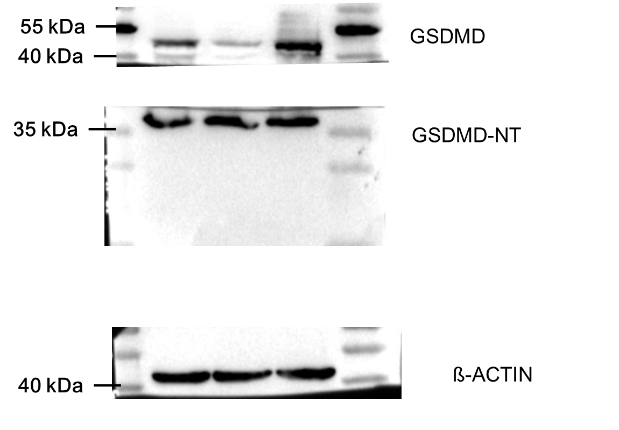

Supplement: Supplementary file 1 — Uncropped western blots [file 41420_2026_3149_MOESM1_ESM.docx]
