# Supplementary material for: Induction of stress granules alleviates programmed cell death induced by lysosomal damage during NK cell cryopreservation
Source: Cell Death Discov. 2026 May 7;12:286. doi: 10.1038/s41420-026-03149-0 (PMC13319456; doi:10.1038/s41420-026-03149-0)
Supplement: Supplementary file 2 — Supplementary Materials-cddisc [file 41420_2026_3149_MOESM2_ESM.docx]

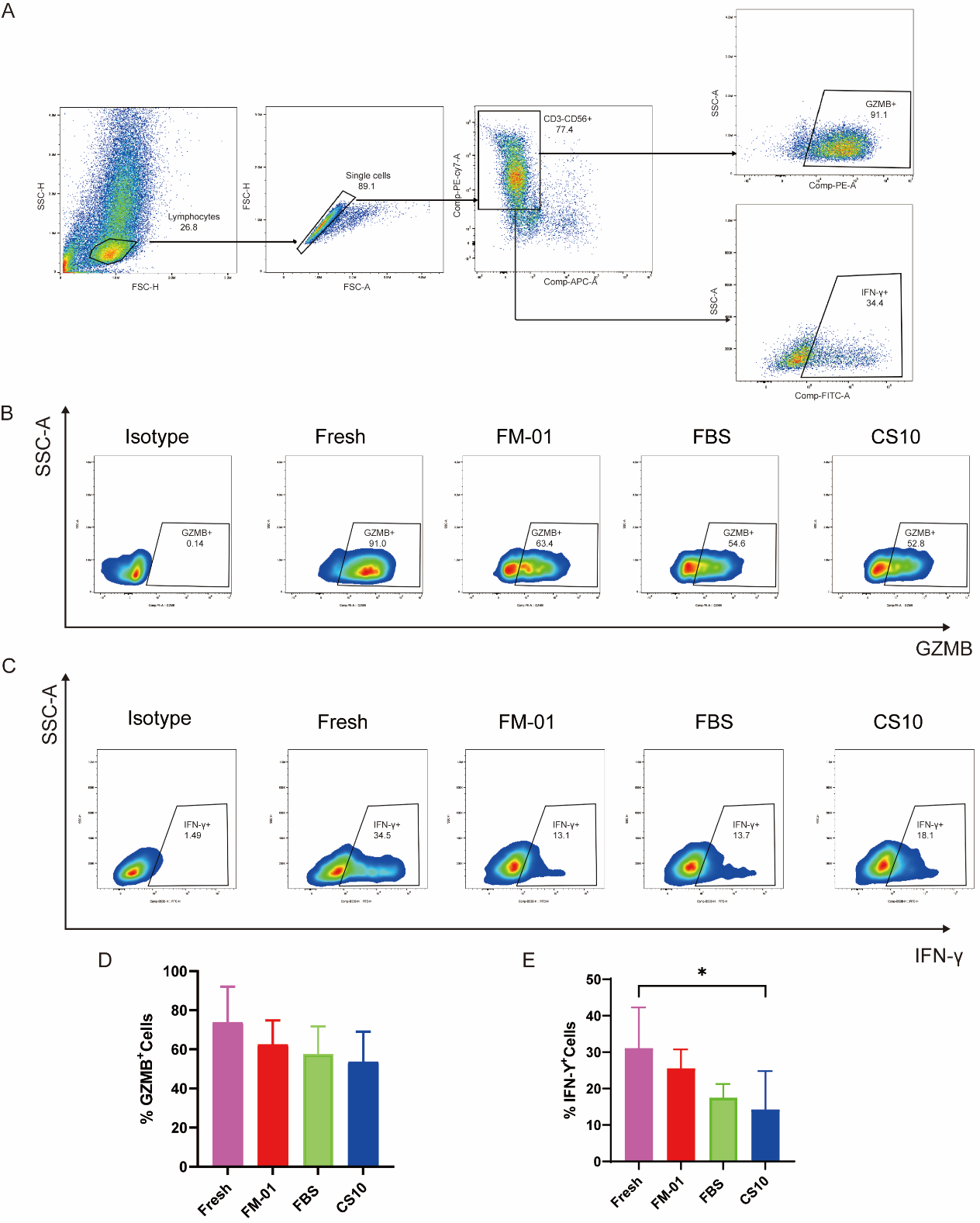


**Supplementary Fig. 1 The cytokine synthesis levels of Fresh and cryopreserved NK cells.** **A** Representative image of gating strategy for GzmB^+^ NK cells and IFN-γ^+^ NK cells by flow cytometry. **B** Representative flow cytometry plot showing expression of GzmB. **C** Representative flow cytometry plot showing expression of IFN-γ. **D, E** Flow cytometry statistics for the expression of GzmB (**D**) and IFN-γ (**E**) (n = 5-7). Data statistics are presented as mean ± SD and were assessed with a one-way ANOVA (D, E) with Tukey’s multiple comparisons test.

**
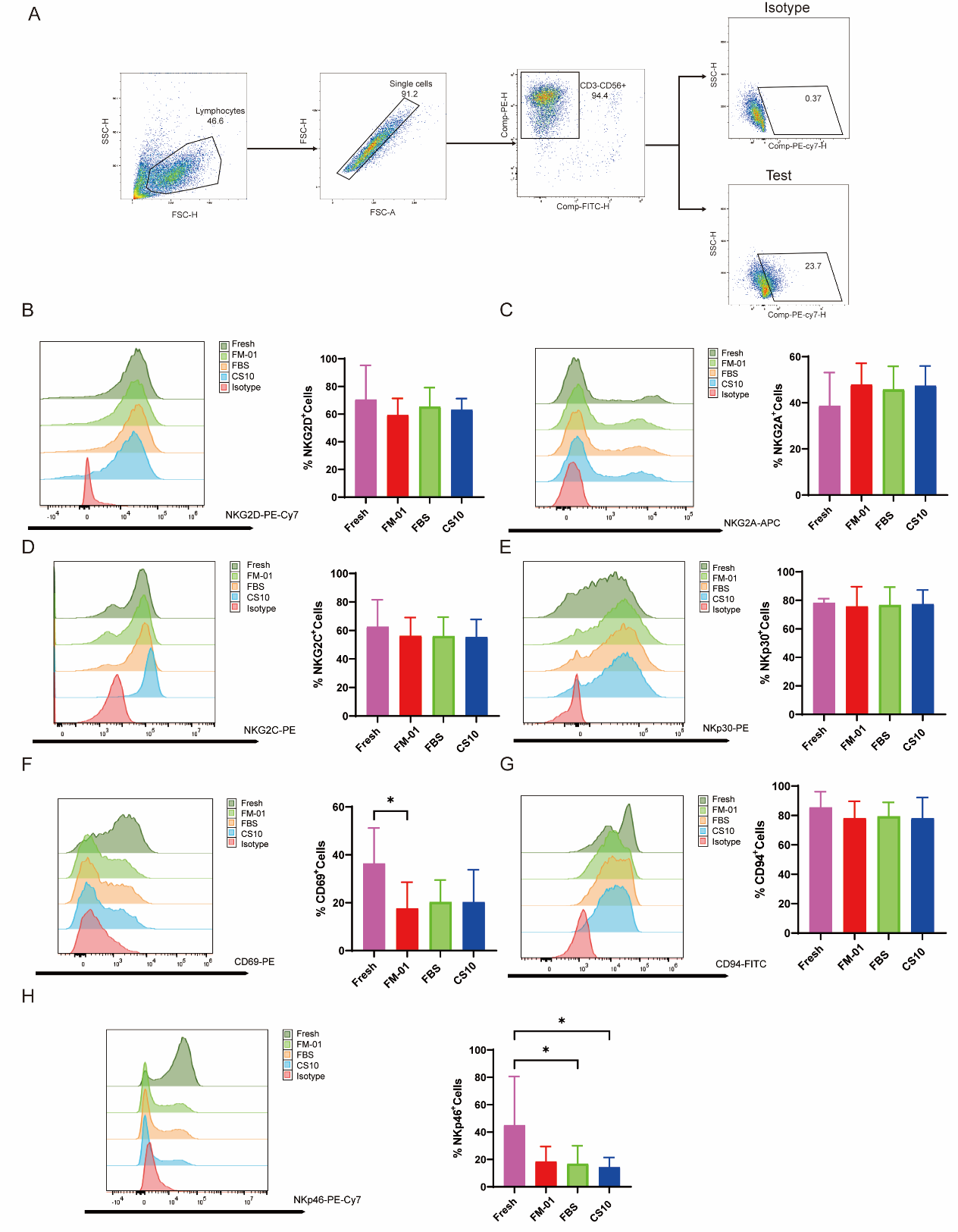
**

**Supplementary Fig. 2 The expression of surface functional receptors and activation markers in fresh and cryopreserved NK cells. A** Gating strategy to analyze the expression of NK cell surface functional receptors and activation markers. Representative flow cytometry histograms (left) and quantitative analysis (right) of (**B)** NKG2D^+^, (**C)** NKG2A^+^, (**D)** NKG2C^+^, (**E)** NKp30^+^, (**F)** CD69^+^, (**G)** CD94^+^, and (**H)** NKp46^+^ in NK cells (n = 7). Data statistics are presented as mean ± SD and were assessed with a one-way ANOVA (B-H) with Tukey’s multiple comparisons test.


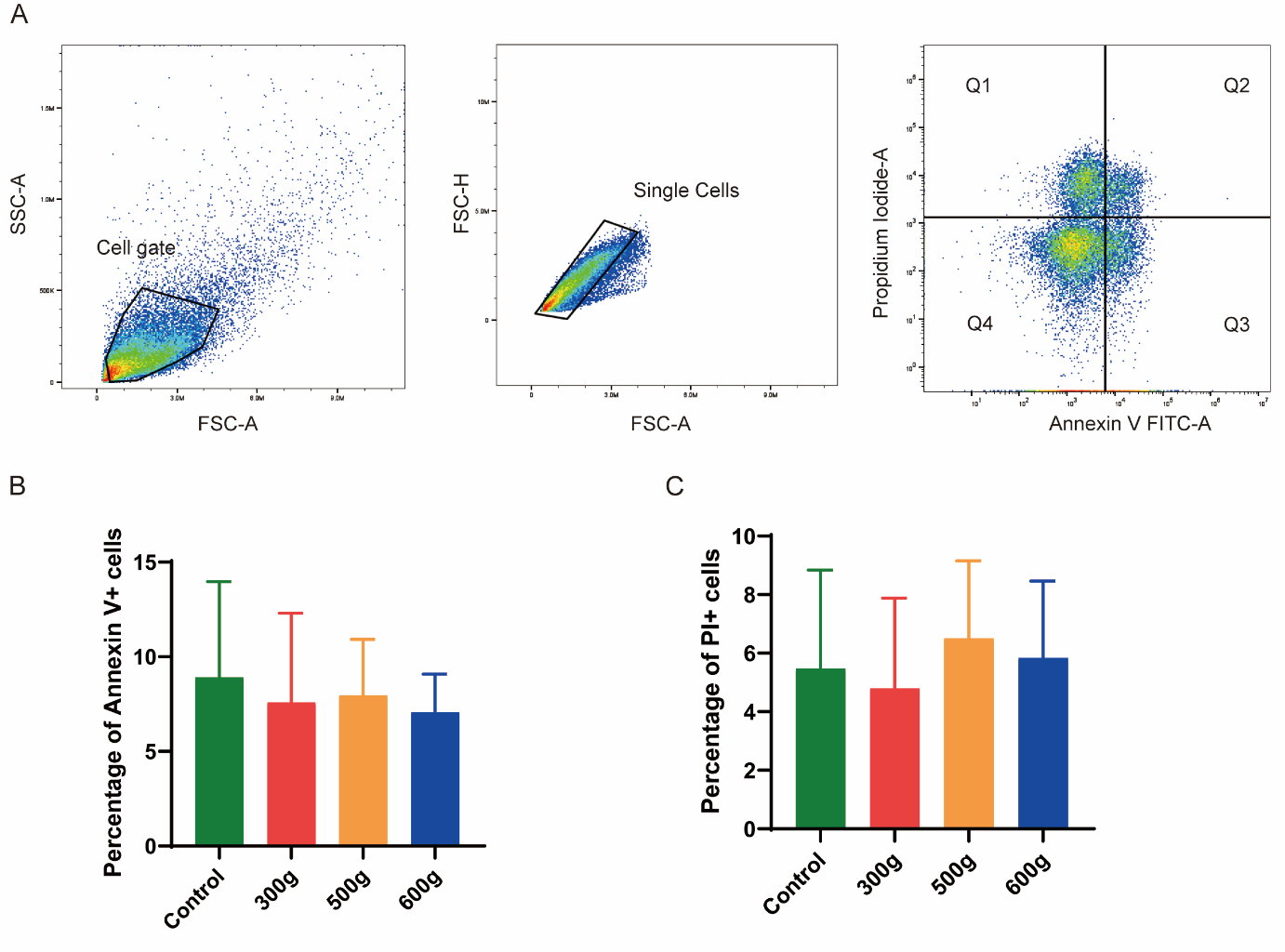


**Supplementary Fig. 3** **Effect of centrifugal speed on apoptosis and necrosis levels in fresh NK cells. A** The gating strategy for the Annexin-V/PI apoptosis analysis. **B, C** Apoptosis (**B**) and necrosis (**C**) levels in fresh NK cells at varying centrifugal speeds (n = 5). Data statistics are presented as mean ± SD and were assessed with a one-way ANOVA (B, C) with Tukey’s multiple comparisons test.

**
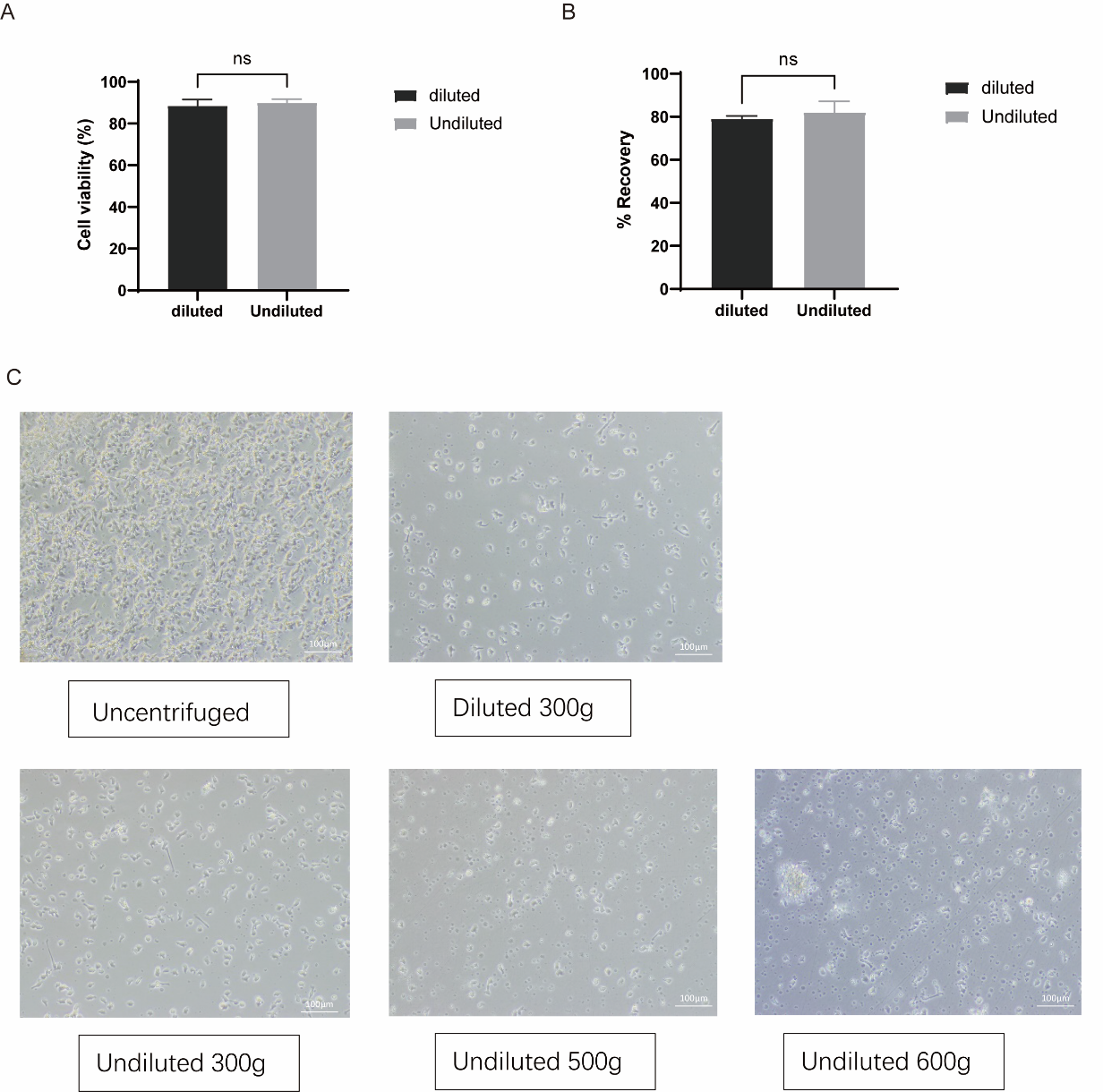
**

**Supplementary Fig. 4 Effects of post-thaw dilution and RCF on cryopreserved NK cells. A** Viability of cryopreserved NK cells immediately after thawing, with or without post-thaw dilution (n = 4). **B** Recovery of cryopreserved NK cells 24 hours post-thaw, with or without post-thaw dilution (n = 4). **C** Optical microscopy images show the effects of dilution and centrifugation processes on NK cell viability at 72 hours post-thaw. Data (A, B) are presented as the mean ± SD; statistical analyses were performed using unpaired Student’s t-test.


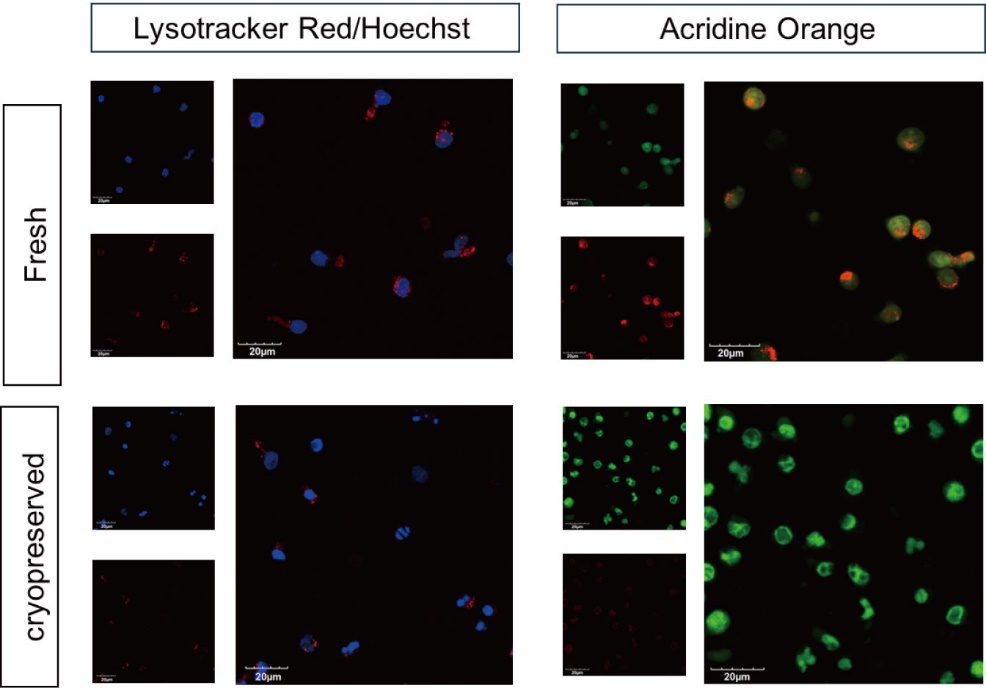


**Supplementary Fig. 5** Fresh NK cells and cryopreserved NK cells (using FM-01 cryomedia) at 0 h post-thaw were incubated with 100 nM LysoTracker Red for 30 min to assess lysosomal stability, or with 2 μg/ml AO for 30 min to evaluate lysosomal dysfunction.

**Supplementary Table 1**

| **Target** | **Conjugate** | **Company** | **Catalog number** |
| --- | --- | --- | --- |
| CD3 | APC | Biolegend | 300312 |
| CD3 | FITC | Biolegend | 300406 |
| CD56 | PE | Invitrogen | 12-0566-42 |
| CD56 | PE-CY7 | Invitrogen | 25-0567-42 |
| CD69 | PE | Biolegend | 310906 |
| CD45 | PE-CY7 | Invitrogen | 25-0459-42 |
| CD94 | FITC | Invitrogen | 11-0949-42 |
| IFN-γ | FITC | Invitrogen | 11-7319-82 |
| GZMB | PE | Invitrogen | 12-8896-42 |
| NKp46 | PE-CY7 | Invitrogen | 25-3359-42 |
| NKp30 | PE | Invitrogen | 12-3379-42 |
| NKG2C | PE | Biolegend | 375004 |
| NKG2A | APC | Biolegend | 375108 |
| NKG2D | PE-CY7 | Invitrogen | 25-5878-42 |
